# Data on whole length myosin binding protein C stabilizes myosin S2 as measured by gravitational force spectroscopy

**DOI:** 10.1016/j.dib.2018.04.002

**Published:** 2018-04-05

**Authors:** Rohit R. Singh, James W. Dunn, Motamed M. Qadan, Nakiuda Hall, Kathy K. Wang, Douglas D. Root

**Affiliations:** Department of Biological Sciences, Division of Biochemistry and Molecular Biology, University of North Texas, Denton, TX 76203, United States

## Abstract

Data presented in this article relates to the research article entitled “Whole length myosin binding protein C stabilizes myosin subfragment-2 (S2) flexibility as measured by gravitational force spectroscopy.” (Singh et al., 2018) [1]. The data exhibits the purified skeletal myosin binding protein C (MyBPC) from rabbit back muscle was of slow skeletal type confirmed by chromatography and in unphosphorylated state based on its isoelectric point (pI) by chromatofocussing. The competitive enzyme linked immunosorbent assay (cELISA) data displayed the site specificity of polyclonal anti-S2 antibody to myosin S2. This polyclonal antibody binding site corresponds to a familial hypertrophic cardiomyopathy (FHC) point mutation hotspot on myosin S2 illustrated in a figure of compiled data.

**Specifications table**

**Table:** 

Subject area	*Biochemistry and Biophysics*
More specific subject area	*Purification of skeletal MyBPC, Site specificity of polyclonal anti-S2 antibody, FHC mutation hotspots along myosin molecule, MALDI-TOF mass spectroscopy*
Type of data	*Figures and Images*
How data was acquired	*Chromatography, Sodium dodecyl sulfate polyacrylamide gel electrophoresis, Enzyme Linked Immunosorbent Assay, Literature Survey*
Data format	*Analyzed, Raw*
Experimental factors	For data 1, skeletal MyBPC was isolated and purified from rabbit back muscle.
	For data 2, site specificity for polyclonal antibody was judged by the competitive binding of the antibody to human myosin S2 cardiac peptide and rabbit skeletal myosin.
	For data 3, FHC point mutations were surveyed from literature and mapped to its amino acid position along the myosin molecule.
Experimental features	*For data 1, skeletal MyBPC was purified sequentially by hydroxyapatite, gel filtration and anion exchange chromatography. The pI of the purified protein was determined by chromatofocusing. Mass and purity was determined by SDS-PAGE.*
	*For data 2, cELISA was performed with two myosin S2 binding sites for polyclonal anti-S2 antibody, one on human cardiac myosin S2 peptide and other on whole rabbit skeletal myosin to confirm the myosin S2 site specificity for the antibody. Purity of antigens was confirmed by SDS-PAGE and mass spectroscopy.*
	*For data 3, the figure was constructed with blue diamonds, indicating the FHC point mutation on myosin molecule.*
Data source location	*University of North Texas, Denton, Texas - 76203*
Data accessibility	*Associated with this article*
Related research article	*Whole length myosin binding protein C stabilizes myosin S2 flexibility as measured by gravitational force spectroscopy.*[1]

**Value of the data**

• Purification of skeletal myosin binding protein C.
• Determination of unphosphorylated state of slow skeletal type myosin binding protein C.
• Site specificity of new polyclonal anti-S2 antibody.
• Familial hypertrophic cardiomyopathy mutations and the antibody binding site.

## 1 Data

The data exhibits the isolation and purification of MyBPC from rabbit back muscles. The data highlights the purified skeletal MyBPC was of slow skeletal type with pI 5.6 and apparent molecular weight of 150 kilo Daltons (kDa). The pI of the purified skeletal MyBPC also confirmed the unphosphorylated state of the protein. The cELISA data confirmed the site specificity for the polyclonal anti-S2 antibody, where myosin S2 on whole rabbit skeletal myosin competed with the human cardiac myosin S2 peptide to bind the polyclonal anti-S2 antibody. The figure data gives the visual aid for the FHC point mutations found across the myosin molecule. The point mutations were compiled from several studies and reviews also highlighting the FHC mutation hotspot found in myosin S2.

## 2 Experimental design, materials, and methods

### 2.1 Purification of skeletal MyBPC (Data 1)

#### 2.1.1 Extraction of MyBPC

Crude extract of MyBPC was obtained from the stored rabbit skeletal myofibrils by the method described by Furst et al. [2]. Crude MyBPC was extracted in a two-step process first by suspending myofibrils in extraction buffer (0.6 M potassium chloride, 2 mM magnesium chloride, 2 mM ethylene glycol-bis(β-aminoethyl ether)-N,N,N′,N′-tetraacetic acid, 1 mM 2-mercaptoethanol, 1 mM sodium azide, 10 mM imidazole, pH 7.0) and centrifuging it to collect supernatant. For the second step, the supernatant was dialyzed against the dialysis buffer (2 mM ethylene glycol-bis(β-aminoethyl ether)-N,N,N′,N′-tetraacetic acid, 1 mM 2-mercaptoethanol, 1 mM sodium azide, 50 mM Tris-hydrochloric acid, pH 7.9). After dialysis, the supernatant collected through centrifugation gave the crude extract of MyBPC. Purification of MyBPC from the crude extract was performed through a series of chromatography techniques.

#### 2.1.2 Hydroxyapatite column chromatography

**Fig. 1.1:**
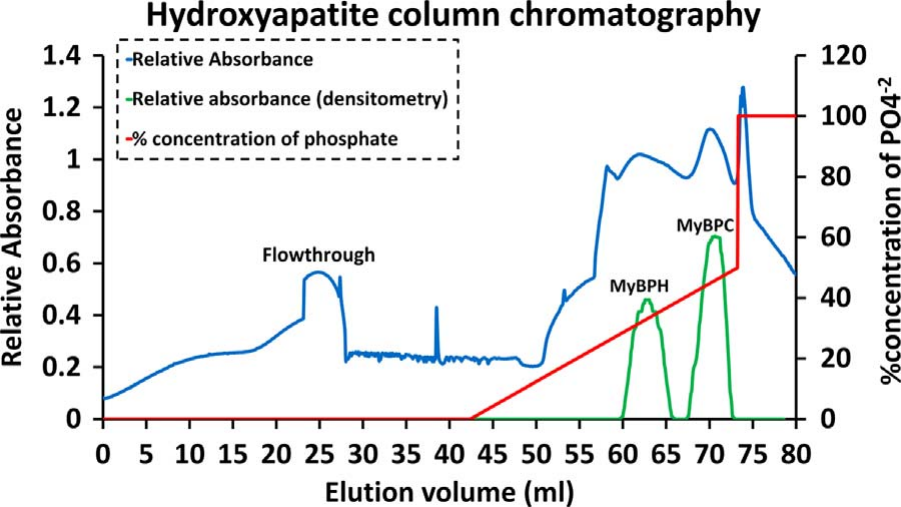
Hydroxyapatite column chromatography of crude MyBPC extract. UV absorbance at 254 nm was read from a flow cell (blue line). SDS-PAGE of the fractions was analyzed by densitometry at mobilities corresponding to myosin binding protein H (MyBPH) and myosin binding protein C (MyBPC) (green line). The 100% phosphate concentration was at a total of 500 mM phosphate.

Hydroxyapatite column chromatography was performed with the crude extract of MyBPC. Proteins were bound to the column, in presence of low phosphate buffer (0.3 M potassium chloride, 4.8 mM potassium phosphate dibasic, 5.2 mM potassium phosphate monobasic, pH 7.0). Proteins bound to the column are eluted by a gradient of high phosphate buffer (0.3 M potassium chloride, 340 mM potassium phosphate dibasic, 160 mM potassium phosphate monobasic, pH 7.0). Proteins are eluted based on the charge, higher the charge carried by protein later is the protein eluted from the column. Fraction with proteins were identified by ultraviolet absorbance of the fractions (Fig. 1.1) and later checked for MyBPC by denaturing or sodium dodecyl sulfate polyacrylamide gel electrophoresis (SDS-PAGE). The MyBPC eluted at over 100 mM higher phosphate concentration than myosin binding protein H (MyBPH) which is consistent with the elution of slow skeletal MyBPC rather than fast skeletal MyBPC which elutes at only 60 mM higher phosphate concentration than MyBPH [3].

#### 2.1.3 Size exclusion chromatography

**Fig. 1.2:**
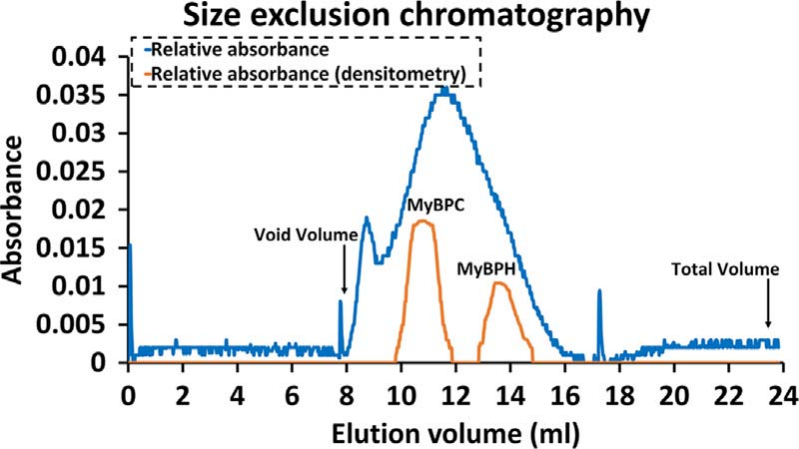
Size exclusion chromatogram of MyBPC fractions. UV absorbance at 254 nm was read from a flow cell (blue line). SDS-PAGE of the fractions was analyzed by densitometry at mobilities corresponding to MyBPH and MyBPC (orange line).

To further purify MyBPC, a size exclusion chromatography was performed with Toyopearl HW-55 column low phosphate buffer as used earlier. Toyopearl HW-55 has a size exclusion limit of 700,000 Da, and MyBPC has the size of approximately 140,000 Da, thus performing this chromatography would get rid smaller molecular weight proteins. The buffer used for size exclusion chromatography was at 10 mM of phosphate buffer at pH 7.0. Fractions collected (Fig. 1.2) were subjected to denaturing polyacrylamide gel electrophoresis to check the presence of MyBPC at 140,000 - 150,000 Da.

#### 2.1.4 Anion exchange chromatography

**Fig. 1.3:**
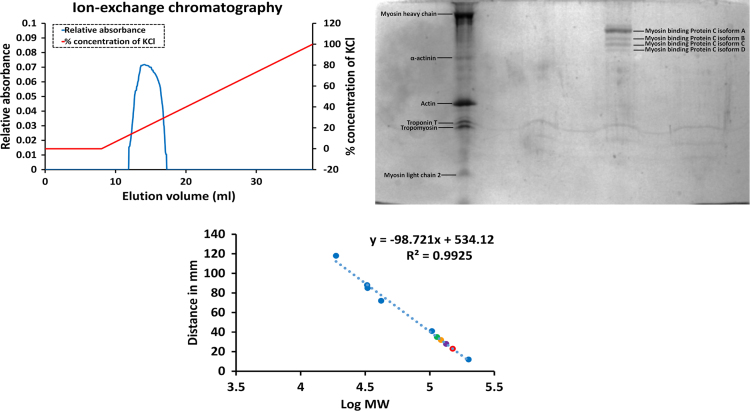
Ion exchange chromatography purification and determination of molecular weight of MyBPC. (1.3.1) Chromatogram of purified MyBPC. (1.3.2) SDS-PAGE gel of purified myosin binding protein C after ion exchange chromatography (1.3.3) Standard curve of distance travelled by standard protein bands versus the log of molecular weight of the proteins with MyBPC isoform A (red plot), MyBPC isoform B (purple plot), MyBPC isoform C (orange plot) and MyBPC isoform D (green plot) plotted.

Anion exchange chromatography was performed with the above size exclusion chromatography fractions containing MyBPC to purify it much further. Diethylaminoethanol linked sepharose column are positively charged and allow protein to bind the column through ionic bonds with low salt buffer (20 mM Tris hydrochloric acid, pH 7.5). Proteins are eluted by running a gradient of high salt buffer (20 mM Tris hydrochloric acid, 1 M potassium chloride pH 7.5). The fractions containing the purified MyBPC was collected based on the chromatogram (Fig. 1.3). Denatured polyacrylamide gel electrophoresis confirmed the purification of MyBPC. The gel picture of the fraction containing MyBPC is imaged in Fig. 1.3.2. Several isoforms of MyBPC were observed in the gel labelled A, B, C and D.

The gene database indicates three isoforms of myosin binding protein C gene namely, skeletal myosin binding protein C slow type (NC_013672, theoretical molecular weight=132.53 kDa and theoretical pI at pH 5.7), skeletal myosin binding protein C fast type (NW_003161100, theoretical molecular weight=127.2 and theoretical pI at pH 6.2) and myosin binding protein C cardiac type (NC_013669, theoretical molecular weight=140.36 kDa and theoretical pI at pH 6.3). The theoretical molecular weight and pI was calculated by ExPASy online tool. Consistent with the reported apparent molecular weight by SDS-PAGE of rabbit slow skeletal MyBPC of 152 kDa [3], the molecular weight of MyBPC isoform A was at 150 kDa with 75% purity followed by MyBPC isoform B at molecular weight 134 kDa with 8.9% purity, MyBPC isoform C at molecular weight 122 kDa with 11.5% purity and MyBPC isoform D at molecular weight 114 kDa with 4.6% purity (Fig. 1.3.3). The MyBPC was extracted from rabbit skeletal back muscles and majority of MyBPC purified belonged to isoform A, which could represent either slow type or cardiac type MyBPC while the isoforms B, C and D could represent the different isoforms of low molecular weight fast skeletal type MyBPC.

#### 2.1.5 Chromato-focusing

**Fig. 1.4:**
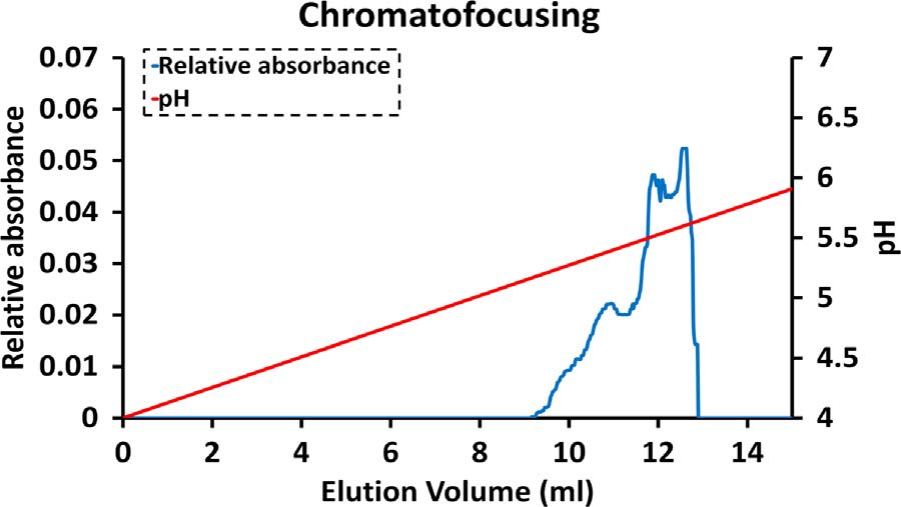
Chromatogram for verification of MyBPC by chromatofocusing. UV absorbance at 254 nm was read from a flow cell (blue line).

The verification of MyBPC isoforms from ion exchange fraction was done by performing chromato-focusing and elution of the protein based on its (pI). Chromato-focusing utilizes the isoelectric point of the protein to isolate the protein. The column used for chromato-focusing was Polybuffer Exchanger 94, and 25 mM imidazole at pH 6.5 was used to load the protein fractions from the previously performed anion ion exchange chromatography. Ten times diluted Polybuffer 74 with a pH 4.2 was used to create a pH gradient from 4.0 to 7.0. The calculated pI of unphosphorylated rabbit slow type skeletal MyBPC (NC_013672) is at pH 5.7 (ExPASy) and pH 5.6 (ANTHEPROT). The protein eluted was at pH around 5.6 (Fig. 1.4) confirming that protein fractions obtained after anion exchange chromatography were indeed unphosphorylated slow type skeletal MyBPC, since phosphorylated MyBPC would have a lower pI [4]. Furthermore, the MyBPC was purified from frozen rabbit skeletal myofibrils and with no additional calcium or kinase treatment to induce phosphorylation of serine residues [5]. Purified MyBPC was further flash frozen in liquid nitrogen and stored at −70 °C in small aliquots for further use.

All the above column chromatography procedures were performed on a Fast Protein Liquid Chromatography system from Pharmacia Biotech. All the columns used were also obtained from Pharmacia Biotech.

Polyacrylamide gel electrophoresis performed to analyze the chromatography fractions were of discontinuous and denaturing type. The 2% stacking gel and 10% resolving gel were prepared by polymerizing acrylamide with linker bisacrylamide with ammonium persulfate, tetramethylethylenediamine and sodium dodecyl sulfate. Stacking gel was prepared in 0.5 M Tris hydrochloric acid buffer with pH 6.8 and resolving gel was prepared in 1.5 M Tris hydrochloric acid buffer with pH 8.8. Running buffer for electrophoresis was made of 25 mM Tris hydrochloric acid, 0.1% sodium dodecyl sulfate, 0.2 M glycine with the pH 8.3. Protein fractions were denatured by boiling the fractions with loading buffer (glycerol, 2-mercaptoethanol, 10% sodium dodecyl sulfate and 0.1% bromophenol blue in 0.5 M Tris hydrochloric acid buffer with pH 6.8). Staining of the gels was done in solution containing 0.25% Coomassie brilliant blue G250 with 1:5:6 acetic acid: methanol: water and destained with solution containing 1:1:14 methanol: acetic acid: water.

### 2.2 Site specificity of posthinge polyclonal anti-S2 antibody (Data 2)

Competitive ELISA was performed to judge the site specificity of polyclonal anti-S2 antibody to myosin S2. cELISA was setup with two binding sites for polyclonal anti-S2 antibody one being myosin S2 on rabbit skeletal myosin and other being human cardiac myosin S2 peptide which was used to obtain the polyclonal anti-S2 antibody. cELISA was performed in a two step process, first, the minimum dilution of polyclonal anti-S2 antibody with assay buffer that would give a positive optical density upon binding to rabbit skeletal myosin S2 coated on to the wells of the microtiter plate. Second was the amount of cardiac myosin S2 peptide would compete with rabbit skeletal myosin S2 to bind polyclonal anti-S2 antibody, thus confirming the site specificity of polyclonal anti-S2 antibody to myosin S2.

The wells of the microtiter plate were coated with approximately, 200 nanograms of rabbit skeletal myosin diluted with cELISA assay buffer; 100 mM potassium phosphate dibasic, 100 mM potassium phosphate monobasic, 1 M potassium chloride and 10 mM magnesium chloride with pH 7.0 overnight. Several dilutions of primary polyclonal anti-S2 antibody were prepared in assay buffer. The primary antibody dilutions created with assay buffer were in the increasing exponential order of 2 for example; 1:2, 4, 8…,512. This series of diluted primary polyclonal anti-S2 antibody were added in replicates to the wells of microtiter plates. After incubation for an hour, the unbound primary polyclonal anti-S2 antibody was washed with phosphate buffered saline (PBS); 0.14 M sodium chloride, 2.7 mM potassium chloride, 1.5 mM potassium phosphate monobasic, and 8.1 mM sodium phosphate dibasic and adjusted to pH 7.4 and blocking buffer; 3% powdered milk in detergent buffer. Enzyme linked secondary antibody diluted 30,000 times in blocking buffer was added to all the wells and incubated at room temperature for an hour.

After incubation, the plates were washed with detergent buffer; 0.05% triton X-100 in PBS, and later BCIP substrate was added. The microtiter plate was placed on a light box next to an optical density calibration scale under a video camera to record the color development in plates. The video was analyzed by Image J to acquire the optical density in the wells. The dissociation constant (*K*_d_) of primary polyclonal anti-S2 antibody to rabbit skeletal myosin S2 was at a dilution titer of 127±4 of diluted primary polyclonal anti-S2 antibody (Fig. 2.1). For optimal OD development, 1:64 dilution of primary polyclonal anti-S2 antibody was used in the following cELISA assay.

**Fig. 2:**
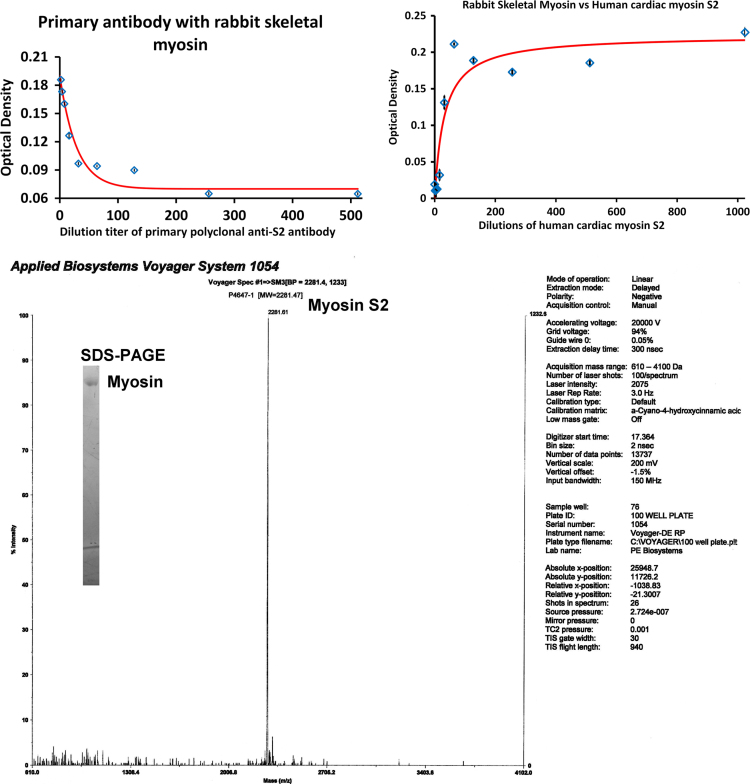
cELISA for site specificity of polyclonal anti-S2 antibody to myosin S2. (2.1) Optical density versus dilutions of primary anti-S2 antibody. Mapped on *x*-axis is the dilution titers for primary polyclonal anti-S2 antibody. (2.2) cELISA for competitive binding of polyclonal anti-S2 antibody to human β-cardiac myosin S2 peptide and rabbit skeletal myosin S2. Mapped on *x*-axis is the dilution titers for human β-cardiac myosin S2 peptide. (2.3) Purity of antigens by MALDI-TOF mass spectroscopy for the myosin S2 synthetic peptide of 2281.5 Da molecular weight and by 10% SDS-PAGE for rabbit skeletal myosin in the inset.

To evaluate the amount of cardiac myosin S2 peptide that would compete with rabbit skeletal myosin S2 to bind primary polyclonal anti-S2 antibody, the microtiter plate was coated with rabbit skeletal myosin first and to that was added two-fold dilutions of 1 µM stock of human cardiac myosin S2 peptide from 1:2 to 1:1024. Later 1:64 diluted primary polyclonal antibody was added to the wells. After the washing step, secondary antibody is added followed by BCIP and development of color or OD was recorded, and the video was analyzed by Image J. The OD will increase as the concentration of human cardiac myosin S2 decreases. With decreasing amounts of human cardiac myosin S2, the primary polyclonal anti-S2 antibody would bind to the next available myosin S2 on rabbit skeletal myosin allowing the enzyme conjugated secondary antibody to bind thus there will be increasing color development upon addition of BCIP substrate.

The minimum amount of human cardiac myosin S2 peptide that would bind primary polyclonal anti-S2 antibody was at a dilution of 1:32 with dissociation constant (*K*_d_) corresponding to a dilution of 32±1 from the stock (Fig. 2.2). Such a high binding affinity was expected, since primary polyclonal anti-S2 antibody was raised against the same human cardiac myosin S2 peptide.

The purity of the antigens was assessed by SDS-PAGE for myosin and MALDI-TOF mass spectroscopy for the synthetic myosin S2 peptide (Fig. 2.3). The SDS-PAGE was performed by the same procedure as that for MyBPC (Fig. 1.3.2).

### 2.3 FHC mutation hotspots in myosin S2 and the posthinge antibody binding site (Data 3)

**Fig. 3:**
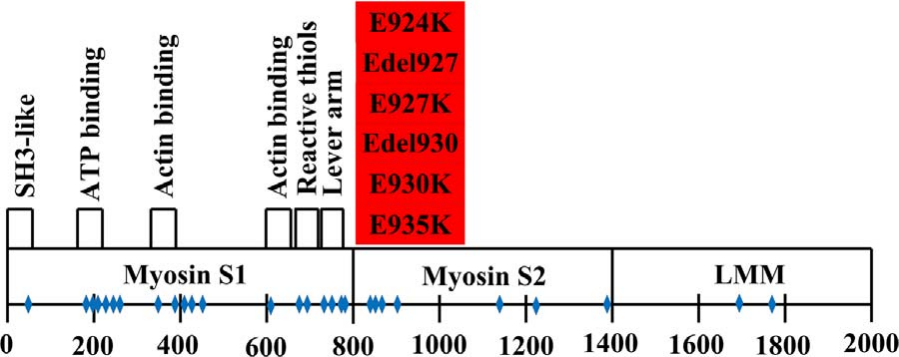
Familial hypertrophic cardiomyopathy point mutations (blue diamonds) across MYH7 gene with point mutations highlighted in the proximal myosin S2 region that correspond to the posthinge region. (The mutations are based on published data including large clinical studies of consecutive patients to provide a representative but not complete illustration of the distribution of familial hypertrophic cardiomyopathy point mutations [10–17].).

Familial Hypertrophic Cardiomyopathy mutations along the myosin molecule length were reviewed. The figure data demonstrates blue diamonds as the point mutation for an amino acid in that region of myosin molecule. The data was collected from several reviews and large clinical studies of consecutive patients [6], [7], [8], [9], [10], [11], [12], [13]. The figure was constructed to highlight the mutation hotspot present in the myosin subfragment-2 region of myosin molecule that is the focus of the site specific polyclonal antibody to the posthinge region ( Fig. 3).
